# Modified Red Blue Vegetation Index for Chlorophyll Estimation and Yield Prediction of Maize from Visible Images Captured by UAV

**DOI:** 10.3390/s20185055

**Published:** 2020-09-05

**Authors:** Yahui Guo, Hanxi Wang, Zhaofei Wu, Shuxin Wang, Hongyong Sun, J. Senthilnath, Jingzhe Wang, Christopher Robin Bryant, Yongshuo Fu

**Affiliations:** 1 Beijing Key Laboratory of Urban Hydrological Cycle and Sponge City Technology, College of Water Sciences, Beijing Normal University, Beijing 100875, China; guoyh@lreis.ac.cn (Y.G.); 202031470020@mail.bnu.edu.cn (Z.W.); 202021470023@mail.bnu.edu.cn (S.W.); 2 State Environmental Protection Key Laboratory of Wetland Ecology and Vegetation Restoration/School of Environment, Northeast Normal University, Jingyue Street 2555, Changchun 130017, China; wanghx197@nenu.edu.cn; 3 The Center for Agricultural Resources Research, Institute of Genetics and Developmental Biology& Center for Agricultural Resources Research, Institute of Genetics and Developmental Biology, The Chinese Academy of Sciences, 286 Huaizhong Road, Shijiazhuang 050021, China; hysun@sjziam.ac.cn; 4 Institute for Infocomm Research, Agency for Science, Technology and Research (A*STAR), Singapore 138632, Singapore; jsenthilnath@alum.iisc.ac.in; 5 MNR Key Laboratory for Geo-Environmental Monitoring of Great Bay Area of the Ministry of Natural Resources & Guangdong Key Laboratory of Urban Informatics & Shenzhen Key Laboratory of Spatial Smart Sensing and Services, Shenzhen University, Shenzhen 518060, China; Jingzhewang@szu.edu.cn; 6 The School of Environmental Design and Rural Development, University of Guelph, Guelph, ON N1G 2W1, Canada; christopher.robin.bryant@gmail.com

**Keywords:** maize, UAV/UAS, chlorophyll contents, yield predictions, machine learning

## Abstract

The vegetation index (VI) has been successfully used to monitor the growth and to predict the yield of agricultural crops. In this paper, a long-term observation was conducted for the yield prediction of maize using an unmanned aerial vehicle (UAV) and estimations of chlorophyll contents using SPAD-502. A new vegetation index termed as modified red blue VI (MRBVI) was developed to monitor the growth and to predict the yields of maize by establishing relationships between MRBVI- and SPAD-502-based chlorophyll contents. The coefficients of determination (R^2^s) were 0.462 and 0.570 in chlorophyll contents’ estimations and yield predictions using MRBVI, and the results were relatively better than the results from the seven other commonly used VI approaches. All VIs during the different growth stages of maize were calculated and compared with the measured values of chlorophyll contents directly, and the relative error (RE) of MRBVI is the lowest at 0.355. Further, machine learning (ML) methods such as the backpropagation neural network model (BP), support vector machine (SVM), random forest (RF), and extreme learning machine (ELM) were adopted for predicting the yields of maize. All VIs calculated for each image captured during important phenological stages of maize were set as independent variables and the corresponding yields of each plot were defined as dependent variables. The ML models used the leave one out method (LOO), where the root mean square errors (RMSEs) were 2.157, 1.099, 1.146, and 1.698 (g/hundred grain weight) for BP, SVM, RF, and ELM. The mean absolute errors (MAEs) were 1.739, 0.886, 0.925, and 1.356 (g/hundred grain weight) for BP, SVM, RF, and ELM, respectively. Thus, the SVM method performed better in predicting the yields of maize than the other ML methods. Therefore, it is strongly suggested that the MRBVI calculated from images acquired at different growth stages integrated with advanced ML methods should be used for agricultural- and ecological-related chlorophyll estimation and yield predictions.

## 1. Introduction

Maize (*Zea mays* L.) is one of the global dominant crops, and the production of this agricultural crop has contributed more than half of the global non-meat calories and more than 70% energy for animals [1,2,3,4,5]. As the global population is expected to increase about 2.2 billion in the coming three decades, the consumption of agricultural food will be substantially increased [6,7,8]. As a staple food, the agricultural yields of maize are closely correlated with food security and are necessary for maintaining social development [9,10,11]. However, the changing climate may have altered the basic climatic condition of maize, which has affected the growth and further influenced the yields, and there is evidence that the negative impacts of climate change on maize will be more apparent and its influence will be more obvious [12,13]. The inevitably changing climate may result in the reduction of agricultural production in each unit of arable land. Therefore, greater agricultural production will be urgently needed, and thus timely monitoring of the growth conditions and making effective adaptions will be of high priority as the growth conditions are closely correlated with agricultural yields. Precisely predicting the yields of maize is necessary for guaranteeing food security. The leaf chlorophyll content is an indicator representing the growth status of crops and it is crucial for agricultural practices [14,15,16]. The precise estimations of leaf chlorophyll contents will be more useful for agricultural applications as chlorophyll a and chlorophyll b have close relationships with nitrogen concentrations, which are relatively important for the growth of agricultural crops [17,18,19]. The leaf chlorophyll contents can be precisely measured using SPAD-502, which is a nondestructive method for diagnosing the nitrogen (N) status of rice (*Oryza sativa* L.) plants to determine the need for fertilizer-N topdressing [20]. Guo et al. assessed maize at different growth stages under different nitrogen levels using a combined spectrometer comprised of the GreenSeeker and a chlorophyll meter (SPAD-502) [21]. The values of maize measured by SPAD were treated as the true values for testing the potential ability of SPOT (satellite for observation of Earth) images [22]. The correlation between SPOT images and SPAD readings was similar to results between aerial images and SPAD readings, indicating that SPOT images may have potential abilities for detecting the chlorophyll levels and nitrogen stress of maize. Meanwhile, it is really a challenge to predict the crop yields in developing countries like China, as the varieties and management practice of crops vary greatly [23,24]. Thus, accurately acquiring the leaf chlorophyll contents and predicting the yields are crucial for agricultural practices, which is of vital importance for stable growth and development of the economy and society.

There have been several commonly used methods for estimating the chlorophyll contents and yield predictions such as the traditional destructive sampling method (DS), simulation models (SM), and remotely sensed vegetation index (VI). All these techniques have both pros and cons. The DS method is quite precise and reliable, but it has disadvantages for it requires repeated contiguous measurements of samples in the field that are time-consuming, and it was found not to be suitable for estimating the chlorophyll contents for a large area [25,26]. SM are another optional way of precisely assessing the chlorophyll contents at a high resolution, but these methods also rely on a high quality of ground sampling data. Alternatively, the VI calculated from remotely sensed data is a non-destructive method that is usually useful for chlorophyll content estimations and crop yield predictions. VI is commonly calculated using two or more remote-sensed bands that are in linear or non-linear combinations, and which are aimed at enhancing the properties of vegetation and the distributions of canopy structural variations [27,28,29]. Combined with evenly distributed, high-quality sampling data, the VI can be adopted to build regression models with the chlorophyll contents of crops. The VI can be seen as the indicator for monitoring the growing conditions, and the precisely confirmed models can be adopted for scale-up estimations. As the state of the art, the unmanned aerial vehicle (UAV) is now widely used for data collection in agricultural- and ecological-related applications, and it can be quickly deployed for acquiring images when needed compared with traditional satellite remote sensing (SRS) [30,31,32,33,34]. The high temporal and spatial images from UAV platforms are better for analysis as the data are not much influenced by the cloud [35,36,37].

Statistical regression methods are widely used for data analysis in remote sensing domains, but traditional models are linear and not appropriate for scenes where the inner connections between variables are non-linear [38]. Thus, the non-linear regression methods such as machine learning (ML) have many advantages in modeling as they can reflect the real non-linear inner correlations. ML is a learning method that is a subset of artificial intelligence (AI), which involves multiple disciplines such as probability and approximation theory, convex analysis, and so on. Based on the learning method, the ML methods can be classified into supervised learning, unsupervised learning, and reinforcement learning. Supervised learning relies on either training neural networks or decision trees, but either method is highly dependent on the knowledge extraction from the training data set. Neural networks use the extracted information to determine and adjust the networks, weights, and parameters, and decision trees adopt the tree that has the most information. When the models are built using a training data set, new results can be easily predicted using the new networks and decision trees based on the new data set. Thus, supervised learning is widely used in remote sensing domains and agricultural and ecological fields as it can achieve relatively high precision when classifying and regressing. The most advanced supervised learning of ML contains the backpropagation neural network model (BP), support vector machine (SVM), random forest (RF), and extreme learning machine (ELM). These models have been widely used for image classifications, recognition patterns, and object detection, and have achieved relatively high precision in classifications and regressions. However, the differences between different models in yield predictions are not well assessed or investigated, and the advantages and disadvantages are unknown or unclear, especially in monitoring crop growth and yield predictions. In this study, we are trying to (1) propose and evaluate the new RGB VI for monitoring the growth of maize; and (2) assess the performance of ML methods (BP, SVM, RF, and ELM) in yield predictions and scaled-up yields of maize.

## 2. Material and Methods

### 2.1. Study Area

**Figure 1:**
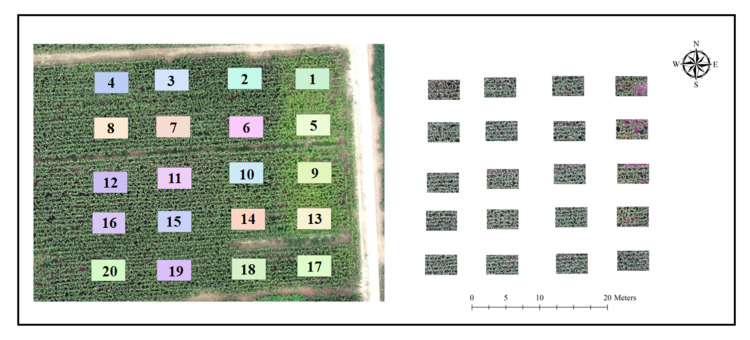
Brief introduction of the study area and sampling plots. Note: The left image represents the whole area and the 20 plots in different colors indicate 20 sampling plots, and the right image represents the corresponding clip subsample images of the 20 plots.

The study area located in the Eco-Agricultural Experimental Station is managed by the Chinese Academy of Sciences (CAS) (Figure 1), and the detailed location of this site is 38.00° N, 116.40° E. The whole area lies in the North China Plain (NCP), which is a main maize and wheat production base of China. To make the most of the total yields of maize and wheat fully utilizing the environment such as sunshine, water, and fertilizer resources, the maize and wheat rotation system (wheat–maize in one year) is adopted as the main management practice in this region. Thus, the yields of maize and wheat can reach the balance and obtain the highest total agricultural yields through this practice. Generally, the growth period of maize will last four months, generally from late June (or early July) to late September (or early October). For the basic climate information, the mean temperature for all year round is 12 ℃ and the mean precipitation is about 480 mm/year.

The study area is located in the semi-humid monsoon climate zone, and the maize is well managed by applying adequate irrigation and sufficient fertilization at important growth stages such as booting (July 21), heading (August 23), and mature (September 15) (Table 1). Nitrogen, phosphorus, and potassium are the main primary nutrients in commercial fertilizers, which play a significant role in plant nutrition and growth. Nitrogen, phosphorus, and potassium are related to the healthiness of plants, the process of photosynthesis, and disease resistance and crop yield increase, respectively. There is a total of twenty sets of comparative experiments through different treatments of fertilizers including different combinations of nitrogenous, phosphate, and potassium fertilizers, and each plot is 10 m long and 8 m wide.

### 2.2. Data Collection

#### 2.2.1. UAV Data Collection

**Figure 2:**
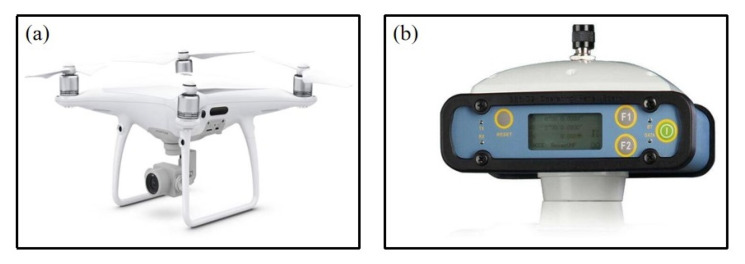
The UAV and RTK system. Note: (**a**) represents the DJI Phantom 4 Pro V2.0; (**b**) represents the RTK S86T system.

In this study, the DJI Phantom 4 Pro V2.0 is adopted as the platform for high-resolution RGB images, with the size of 5472 × 3648 pixels for a single image and a focal lens of 8.8 mm (Figure 2a). Considering the relative flatness of the experiment site, four ground control points (GCPs) are made using white paint before the flight missions of the UAV to obtain the accuracy of the plane geometric. The precise locations of these GCPs are measured by the real-time kinematic (RTK) S86T system and are used for the mosaic of the RGB images (Figure 2b).

To avoid disturbance such as the impacts from different imaging angles of sunlight, all the flight missions were conducted around 10:30 am and the flight altitudes were set as 50 m during the observation of the growth of maize using UAV. To ensure the quality of the data and to try to reduce the effects from the image mosaic, the forward lap of flight was set as 85% and the side lap was set as 80% in Altizure (V4.7.0.196, https://www.altizure.com/my/project), which is a commercial software that is easily deployed for flight control and flight guidance. The long time series of observations using the UAV remote sensing technique were applied, covering the experimental plots. There was a total of eleven flight missions carried out on July 7, July 14, July 22, July 28, August 18, August 25, September 1, September 7, September 14, September 21, and 30 September 2019. All flights covered the GCPs, which were used as a reference for the image mosaic to obtain the same region of interest and to make sure all mosaic images acquired during different dates were all comparable. Finally, the mosaic images of different growth stages were processed and the ground sampling distance (GSD) was 0.018 m for all images.

#### 2.2.2. Ground Measurements

The collections of the chlorophyll contents of maize in each plot measured using SPAD-502 (https://www.specmeters.com/nutrient-management/chlorophyll-meters/spad/) were strictly performed on the same day shortly after conducting the flight missions. For the measurements of chlorophyll contents, the SPAD-502 was applied using the five-points method (FPM) under a standard procedure, including the center and four corners of each plot. The chlorophyll contents of maize for each plot were obtained using the averaged values measured from SPAD-502 with the FPM. For the final yields of each plot, the maize was harvested at the mature stage and the dry matters of each plot were collected and weighed, respectively. Thus, the chlorophyll contents at eleven different dates and the yields after the harvest of each plot were obtained in this way.

### 2.3. Methods

#### 2.3.1. Assessment of the Modified Visible Vegetation Index

VI is commonly used as an important indicator that represents and interprets the growth of vegetation. The VI values were generally used for monitoring the growth and predicting the yields of agricultural crops. The proposed modified red blue vegetation index (MRBVI) was implemented and compared with seven widely used VI approaches that were previously adopted to monitor the growth and to predict the yield of maize. Initially, the RGB bands were converted into normalized forms using the following Equation (1).

R = r ÷ (r + g + b), G = g÷ (r + g + b), B = b÷ (r + g + b)(1)

where r, g, and b are the original digital values from the RGB images. Then, the original values are converted into values that range from 0 to 1, so that the normalized values can better represent the quantitative analyses in remote sensing domains.

E1–E8 were used to calculate the VI that was used to monitor the growth condition and to predict the yields of maize (Table 2). For monitoring the growth conditions of maize, the VI at each stage was calculated and the average values were obtained. The linear relationships function built in Matlab 2019b was used to understand the relationships between the average VI and the corresponding chlorophyll contents measured by SPAD-502 at each plot, and then the coefficient of determination (R^2^) was obtained. The R^2^s for different VIs at different growth stages were acquired and compared with each other. The difference in R^2^ showed the regression ability of the VI, and the higher the R^2^, the more precise the results will be. For the ability of VIs to predict the yields of maize, the VIs were calculated using the subsamples of each plot at different growth stages, and the linear relationships between the average of the VIs and the corresponding yield of maize in each plot were assessed and evaluated.

To better understand the ability of each VI in monitoring the growth of maize at different stages, the VIs and normalized chlorophyll contents measured by SPAD-502 were analyzed together. The change in the VI that was closest to the change in the normalized SPAD values was the best that can be used to describe and monitor the growth conditions of maize.

#### 2.3.2. Yield Predictions of Maize Using ML Methods

**Figure 3:**
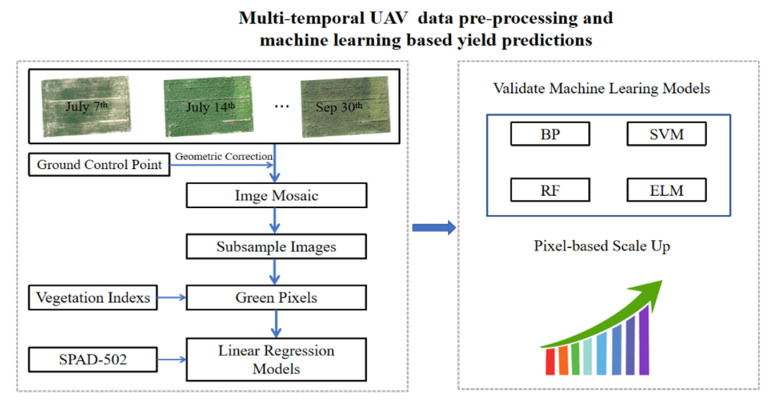
The flow diagram of ML-based yield predictions. The upper part is the multi-temporal images analysis, and the lower part is the ML-based yields predictions.

The detailed flow diagram of image processing and ML-based yield predictions are clearly described (Figure 3). The main work can be divided into two parts: multi-temporal images and processing, and the ML-based yield predictions using a scale-up method. For the multi-temporal images and processing, the images acquired from 11 different dates were spliced together with precision locations of GCPs in the Pix4d mapper under standard procedures. For the mosaic image from each single date, the regions of interest (ROIs) of twenty plots were made using the function built using the ENVI 5.3 software. The twenty ROIs were used to extract the subsample images and there was a total of 220 (11dates × 20plots = 220) subsample images. Then, the subsample images were used to extract pure green pixels using the (EXG-EXR) method, where the EXG equals (2G-R-B). In this way, the subsample images were classified into two categories: green pixels and non-green pixels containing the disturbing background such as soil. The values of the eight VIs of the subsample images using only green pixels were calculated, and the linear relationships between chlorophyll contents, yields of maize, and VIs were built and the R^2^s were obtained. The outperforming VIs calculated using these images during important growth stages were adopted for further analysis of growth monitoring and yield predictions.

Recently, ML has outperformed and showed great potential ability in various applications such as object detection, image classification, recognition patterns, computer visions, and other domains. In supervised learning, the sample data can be divided into training and testing sets, of which the training samples are used to build the non-linear relationships between independent and dependent variables, and the test samples are adopted to assess the effectiveness of trained models. ML methods such as BP, SVM, RF, and ELM have been assessed and evaluated by many studies in remote sensing domains. In this study, the eight VIs calculated from images of the long time series of each plot were set as independent variables and the yields of maize at each plot were set as dependent variables. Each ML model was independently built using the LOO to assess and evaluate the model performance using root mean square error (RMSE), mean absolute error (MAE), and absolute error (AE), which were adopted and calculated. The RMSE and MAE were defined in the following Equations (2)–(4).

(2) $$RMSE=\sqrt{\frac{1}{n}\sum_{1}^{n}{\left(P_{i}-M_{i}\right)}^{2}}$$

(3) $$MAE=\frac{\sum_{i=1}^{n}\left|P_{i}-M_{i}\right|}{n}$$

(4) $$\mathrm{AE}=\sum_{i=1}^{n}\frac{\left|P_{i}-M_{i}\right|}{M_{i}}$$

where *n* is the number of all samples, and *M* and *P* are the true values and predicted values of yields, respectively. The $\bar{M}$ represents the average values of *M*, and $\bar{P}$ is the average values of *P*.

## 3. Results

### 3.1. Assessment of New Vegetation Index in Regression of Chlorophyll Contents

**Figure 4:**
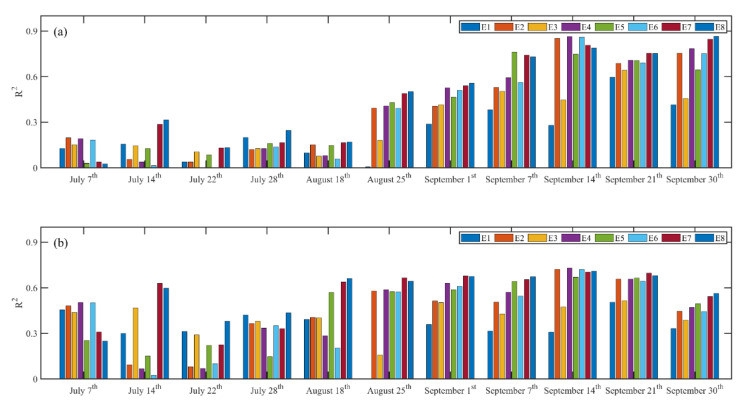
The R^2^ between VI, chlorophyll contents, and yields of maize during the whole growth stages. Note: E is short for equation according to Table 2, and E8 is the MRBVI. (**a**) The R^2^ between VI and chlorophyll contents increased gradually from August 25 (**b**) R^2^ between VI and yield increased significantly with the growing stages of maize, and it especially increased dramatically from August 18.

The coefficients of determination between chlorophyll contents, yields of maize, and VIs were calculated as shown in Figure 4. The R^2^ between VI and chlorophyll contents increased gradually from August 25 (Figure 4a), and the R^2^ between VI and yield increased significantly with the growing stages of maize, and it especially increased dramatically from August 18 (Figure 4b). Thus, it can be found that both R^2^s increased significantly with the growing stages of maize except the early three stages (July 7, July 14, July 22). The VI calculated using images acquired from July 28 to September 30 can be used for yield predictions. Both the R^2^s calculated between VI, chlorophyll contents, and yields of maize were larger than 0.8, therefore it can be indicated that the VI was closely correlated with chlorophyll contents measured using SPAD-502 and yields of maize.

**Figure 5:**
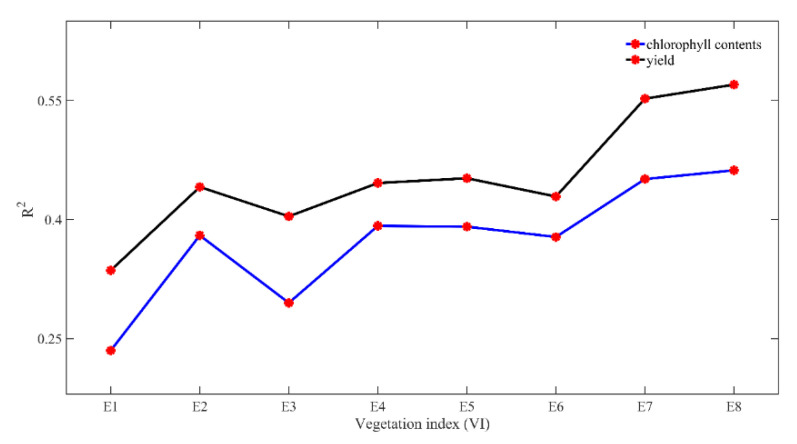
The average of R^2^ between VI, chlorophyll contents, and yields of maize. Note: E is short for equation according to Table 2, and E8 refers to the MRBVI.

The average of R^2^ between VI and chlorophyll contents at 11 different growth stages was 0.235, 0.380, 0.295, 0.392, 0.391, 0.378, 0.451, and 0.462 for E1, E2, E3, E4, E5, E6, E7, and E8, respectively (Figure 5). Similarly, the average of R^2^ between VI and yields from 11 different dates was 0.336, 0.441, 0.404, 0.446, 0.452, 0.429, 0.552, and 0.570 for E1, E2, E3, E4, E5, E6, E7, and E8, respectively. Thus, the proposed MRBVI obtained the highest precision, and it outperformed the other VI approaches, which indicated that the proposed MRBVI has the ability to accurately estimate chlorophyll contents and predict the yields of maize.

**Figure 6:**
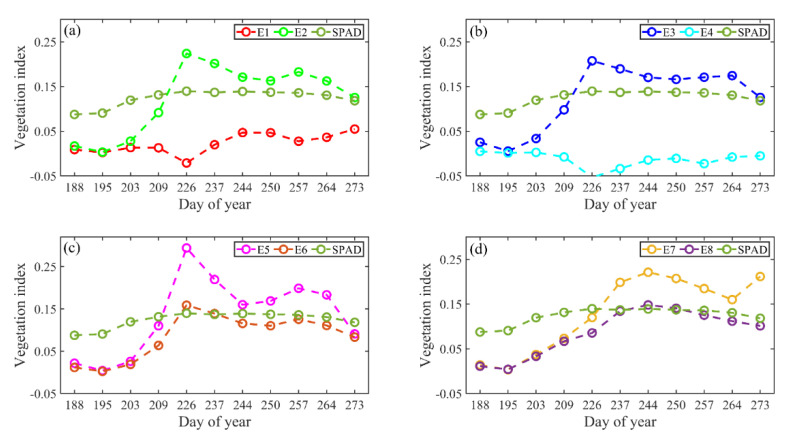
Comparison of VI and normalized SPAD values during the growth of maize. The x-axis indicates the dates of data acquisition in relation to the day of the year. Note: E is short for equation according to Table 2, and E8 is the MRBVI. (**a**) dynamic change of E1, E2 and SPAD; (**b**) dynamic change of E3, E4 and SPAD (**c**) dynamic change of E5, E6 and SPAD and (**d**) dynamic change of E7, E8 and SPAD.

To better show the ability of MRBVI in monitoring the growth conditions during the growth stages of maize, the calculated averages of the existing VIs in addition to our proposed MRBVI of the 20 plots are shown in comparison with the normalized SPAD values measured by SPAD-502 (Figure 6). The MAEs were 0.186, 0.097, 0.089, 0.252, 0.117, 0.078, 0.118, and 0.072, and the AEs were 9.037, 4.978, 4.582, 12.031, 5.838, 4.240, 6.004, and 3.908 for E1, E2, E3, E4, E5, E6, E7, and E8, respectively. Both the MAE and AE of the MRBVI were the smallest, thus it can be observed that the MRBVI had precisely captured the dynamic change of the normalized SPAD values and further indicated the potential ability of MRBVI in chlorophyll contents estimations. Thus, the MRBVI can be deemed as a successful indicator in monitoring the growth conditions of maize when compared with the other seven commonly used VIs.

### 3.2. Prediction of Yield Using ML Methods

**Figure 7:**
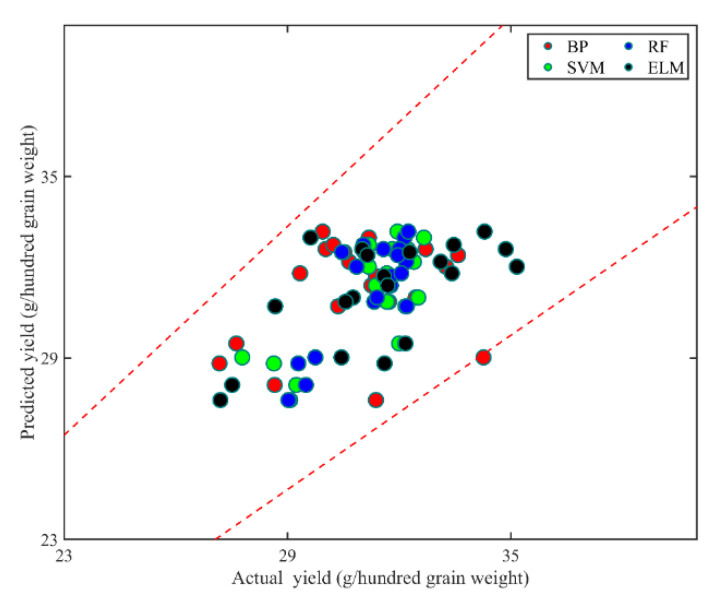
The actual and predicted yield of maize using ML methods, with the red, green, blue, and black nodes representing the results obtained using BP, SVM, RF, and ELM, respectively.

Eight VI approaches were used to calculate the images acquired from dates from July 28 to September 30 and then used to build non-linear ML models with the yields of maize at each plot. These VI values calculated using sub-extracted images were set as independent variables and the yield at each plot was set as the dependent variable. The commonly used BP, SVM, RF, and ELM were adopted for building models using the LOO method. All ML models were independently trained and the RMSE and MAE were used to validate and test the model performance. The predicted yields of maize were shown compared with the actual yields in the scatterplot, and the 15% error lines were added to show the precision of the model performance (Figure 7). It can be intuitively acknowledged that all ML models performed at relatively high precision levels, more specifically, SVM, RF, and ELM performed better than BP as there was one scatter point that fell on the 15% error line. The RMSE and MAE were calculated to assess the performance of the different ML models, and the calculated RMSEs were 2.157, 1.099, 1.146, and 1.698 (g/hundred grain weight) for BP, SVM, RF, and ELM, respectively. Similarly, the calculated MAEs were 1.739, 0.886, 0.925, and 1.356 (g/hundred grain weight) for BP, SVM, RF, and ELM, respectively. Among all methods, SVM had reached the highest precision level in yield predictions, followed by RF, ELM, and BP. Since the four ML models all performed quite well for yield predictions, all models were used to predict the yields of maize combining the scale-up method.

**Figure 8:**
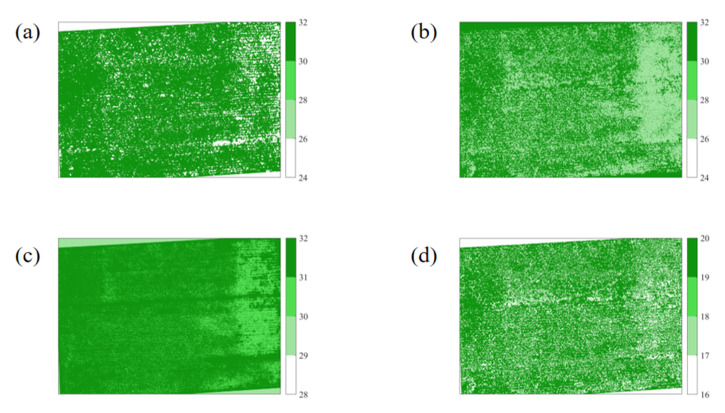
The ML-based yields prediction of maize using the scale-up method. Note: (**a**), (**b**), (**c**), and (**d**) represent the results using BP, SVM, RF, and ELM, respectively.

Since the built models are stable and all the training samples are used for building ML models that can improve the accuracy of models by combining the scale-up methods, the four ML models were well-applied on the pixel-based VI calculated from July 28 to September 30, and were set as independent variables to be put into the ML models for simulations (Figure 8). The pixel-based results in Figure 8 are in accordance with Figure 1, in which Figure 8 contains all 20 plots in Figure 1. The difference in the yields prediction of maize was obvious with the different ML models. The results using BP, SVM, and ELM were likely closer, and the result using RF showed more uncertainties than the other three models even though the RMSE and MAE of RF were both lower than those of BP and ELM. Overall, all ML methods had achieved relatively better performance. In particular, the results using the SVM model were closest to the actual yields using the experimental measurement.

## 4. Discussion

### 4.1. Potential Ability of Modified Vegetation Index

The VI had great potential in assessing and monitoring the growth conditions of crops and predicting the yields. All VI approaches performed better in monitoring the growth and predicting the yield of maize, which is in accordance with previous studies where the VI had been proven to have great potential abilities in agricultural and ecological applications [44,45,46,47]. The MRBVI performed relatively better than the other VIs, and this can be ascribed to the fact that the structure function of the MRBVI emphasized the response of the red band, and it was believed to have a higher correlation with chlorophyll contents than the green and blue bands [48,49]. The proposed MRBVI had the closest relationships with chlorophyll contents measured using SPAD-502, and this was in agreement with previous studies that had found that the red band had greater sensitivity to the chlorophyll contents [48,49]. Thus, the extraction of the conditions of the growth of maize was improved by adding the content of the red band, which was believed to have a higher correlation with chlorophyll contents than the green and blue bands. Thus, the modified VI showed great potential abilities in estimating the phenological variables during the important growth stages of maize as it was closer to the measured chlorophyll contents than the other VIs.

The VIs from different growth stages were the indicators representing the real-time growth conditions (chlorophyll contents). Figure 4 shows that the relationships between VI, chlorophyll contents, and yields varied during the whole growth period, and the R^2^s from the later eight stages were commonly larger than the earlier three stages. This was because the initial stage of maize belongs to the seedling periods when the maize does not cover the whole plots. Thus, the results may be influenced by the background such as soil and other disturbances even though methods had been adopted to eliminate the impacts [39,50,51]. Thus, the integrated VI calculated from UAV RGB images acquired at important growth stages of maize can reflect more precisely the temporal dynamic changes of growth conditions, which can achieve the highest precision for yield prediction [52,53].

### 4.2. Uncertainty and Limitations Using ML Methods

The ML methods are widely used in remote sensing-related domains such as image classification, object detection, and spatial and temporal predictions. There had been reported improvements of ML algorithms, and the methods used in this study were in accordance with the previous applications where the ML methods were successfully applied and conducted [54,55,56,57,58]. Overall, it can be acknowledged that the results from SVM were more precise than those provided by the BP, RF, and ELM models. SVM can be perfectly adopted for processing with increasing variables of input data, especially when the dimensions of training samples exponentially increase from a low dimension to high dimensions [59,60]. The kernel function is applied in SVM, and then the features can be easily converted from low to high dimensions and retain the actual classification effects in high dimensions [61,62]. Through this transformation, the complex calculations of high dimensions can be perfectly avoided [63]. Followed by SVM, RF can also handle the high dimension of data without a dimensionality reduction. RF can judge the importance of the interactions between different features without making feature selection which is time-consuming [64,65,66]. It can still retain high precision when part of the features is lost. RF has a limitation though, as it commonly results in the overfitting phenomenon, which will cause noisy classification and regression problems, making the predictions or simulation unreliable [67]. Originally, BP used an algorithm with signal forward propagation and error backpropagation, which can solve the hidden layer connection weight learning problem of multi-layer neural networks [68,69]. BP has a limitation in that the slow gradient-based learning algorithms are extensively used to train neural networks and all the parameters of the networks are tuned iteratively by using such learning algorithms [70,71]. The advantage of ELM compared to BP is that the connection weight of the input layer and the hidden layer and the threshold of the hidden layer can be randomly set, and there is no need for them to be adjusted [72,73]. Meanwhile, the connection weights between the hidden layer and the output layer were confirmed by solving equations and there was no need for them to be adjusted iteratively. In short, the advantage of ELM was that it was faster than the BP algorithm under the premise of ensuring learning accuracy. The cost times of BP and ELM were assessed and total times for BP and ELM were 355.250 and 332.860 s, respectively. Thus, the estimation of scaling-up using ELM was about 7% faster than BP with higher precision. The RMSE and MAE of BP were 2.157 and 1.739 (g/hundred grain weight), and the RMSE and MAE of ELM were 1.698 and 1.356 (g/hundred grain weight), respectively. Thus, ELM was faster and also had higher precision than BP in the yield predictions.

Overall, the ML models had been successfully conducted for yield predictions of maize. SVM combined with the long time series of the VI should be paid more attention as it had achieved the highest precision among all VIs. Further, the more complicated structure using the iterations of ML models should be of equal importance. For example, the results from one ML model were set as the input of another ML model. Evaluating the different combinations of ML models would achieve higher precision than a single ML model. Deep learning (DL) algorithms have been successfully applied in various aspects in remote sensing and have contributed to domains such as image classification, object auto-detection, image fusion, and registration [74]. The supervised DL model commonly requires a greater number of training data and it has more layers and depth than ML [75]. DL can be used in monitoring the growth conditions of maize and yield predictions as it can obtain higher precision [76]. Thus, integrated ML, DL, and crop models for data assimilation would attract more attention as they will combine the advantages of mechanistic crop models and the advantages of non-mechanical relationships.

## 5. Conclusions

In this study, the performance of the newly proposed MRBVI was assessed and compared with the other seven commonly used VIs for monitoring the growth conditions of maize. The MRBVI performed better as it enhanced the extraction of the red band that was very sensitive to the chlorophyll contents. The eight VIs calculated using multi-temporal images acquired at important growth stages were adopted for predictions of yields for maize using ML methods. The advanced ML methods can achieve a high precision of yield predictions due to the excellent inbuilt computational efficiency and perfect generalizations. The SVM model obtained the highest accuracy with an excellent structural design compared with the other models. Thus, this highlights the necessity of combining the MRBVI calculated from important growth stages and ML methods for the yield prediction of maize.

## Figures and Tables

**Table 1:** The setup of the experiment for maize. The number represents the plots in Figure 1. The abbreviations are listed as: nitrogenous fertilizer (N), phosphate fertilizer (P), potassium fertilizer (K), straw (S), and organic fertilizer (O). The different combinations of fertilizers were applied in each plot. The numbers after fertilizers are the amount of applied fertilizers.

	Column One	Column Two	Column Three	Column Four
Row one	N2S1 (4)	N3P3K1 (3)	N3P1K1 (2)	N1P1K2 (1)
Row two	N2O1 (8)	N3P2K1 (7)	N3P3K2 (6)	N1P1K1 (5)
Row three	N3S1 (12)	N4P3K1 (11)	N2P2K2 (10)	N1P2K1 (9)
Row four	N3O1 (16)	N4P2K1 (15)	N2P1K1 (14)	N1P3K1 (13)
Row five	N4P2K2 (20)	N4P1K1 (19)	N2P2K1 (18)	N2P3K1 (17)

**Table 2:** The commonly used vegetation indices (VIs) and proposed modified red blue VI (MRBVI). Note: E stands for equation and the value k = 0.667; and E8 is the MRBVI.

Index	Equation	Name	Reference
E1	1.4 × R − G	EXR	[39]
E2	(G − B) / (G + B)	NGBDI	[40]
E3	G/(R^k^ × B ^(1−k)^)	COM1	[41]
E4	1.4 × B − G	EXB	[39]
E5	(G − B) / (R − G)	WI	[42]
E6	(R − B) / (R + B)	IKAW	[40]
E7	0.441 × R − 0.811G + 0.385B + 18.787	CIVE	[43]
E8	(R^2^ − B^2^) / (R^2^ + B^2^)	MRBVI	New
